# Infection With *Mycobacterium chelonae* Following Autologous Facial Fat Grafting: A Case Report and Review of Literature

**DOI:** 10.1111/jocd.70631

**Published:** 2025-12-25

**Authors:** Ilad Alavi Darazam, Mahdi Isazadeh, Shirin Zaresharifi, Farahnaz Bidari, Reza M. Robati

**Affiliations:** ^1^ Department of Infectious Diseases and Tropical Medicine, Loghman Hakim Hospital Shahid Beheshti University of Medical Sciences Tehran Iran; ^2^ Skin Research Center Shahid Beheshti University of Medical Sciences Tehran Iran; ^3^ Department of Pathology, Loghman Hakim Hospital Shahid Beheshti University of Medical Sciences Tehran Iran

**Keywords:** atypical mycobacterium, fat grafting, fat transfer, *Mycobacterium chelonae*

## Abstract

**Background:**

Autologous fat grafting is commonly used in cosmetic dermatology, but it can cause serious complications, including nontuberculous mycobacterial infections when done under unsterile conditions.

**Case Presentation:**

We report a 39‐year‐old woman who developed multiple discharging facial abscesses 1 month after undergoing autologous fat grafting in an unauthorized cosmetic clinic. Initial empiric oral therapy with clarithromycin, minocycline, and rifampin failed, prompting intravenous antibiotic therapy. During the hospitalization, she developed loss of consciousness, seizure, cerebral edema, and cavernous sinus thrombosis, followed by corticosteroid‐induced adrenal suppression. When visiting our clinic, biopsy, PCR, and culture were performed, which confirmed infection with *Mycobacterium chelonae* ( *M. chelonae* ) after prolonged multidrug therapy with intravenous amikacin, imipenem, linezolid, and clarithromycin, and abscess drainage; the infection resolved. At 6 months of follow‐up, no recurrence was observed, although residual scarring persisted.

**Conclusion:**

This case highlights a rare and severe systemic complication of *Mycobacterium chelonae* infection following cosmetic fat grafting, underscoring the need for strict aseptic technique, awareness of regional antimicrobial resistance, and better regulation of aesthetic procedures.

## 1 Case Presentation

**FIGURE 1:**
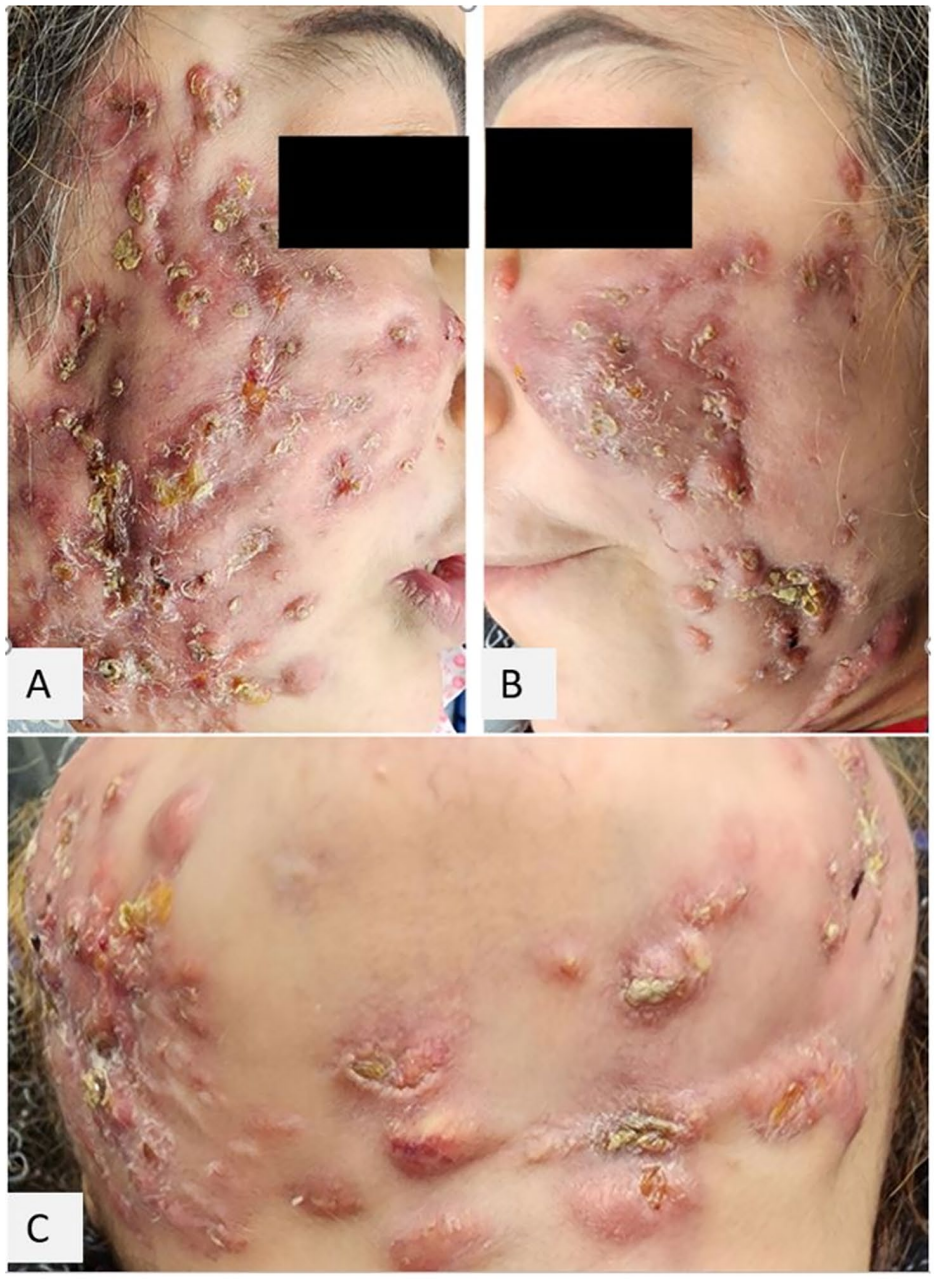
A 39‐year‐old woman presented with multiple nodulocystic lesions on the face accompanied by discharge.

A 39‐year‐old woman presented to our dermatology clinic with multiple discharging nodulocystic lesions on the face. Upon examination, lesions were observed on the cheeks, chin, and submental region, characterized by yellowish discharge and crusting (Figure 1). The patient reported undergoing autologous fat grafting to the face at an unauthorized center in August 2023. One month after the procedure, the patient developed painful, purulent abscesses, necessitating hospitalization for 1 month to manage the infection, during which she received antibiotic therapy.

Approximately 3 months after discharge and resolution of the initial infection, the symptoms recurred, leading to her readmission to the hospital in December 2023. During this second hospitalization, she developed loss of consciousness (LOC), seizures, and cerebral edema.

She was transferred to the intensive care unit (ICU). Clinical evaluation, magnetic resonance imaging (MRI), and magnetic resonance venography (MRV) confirmed cavernous sinus thrombosis, and the patient remained in a coma for 45 days.

Ultimately, after successful management of the infection and cavernous sinus thrombosis and stabilization of her vital signs, she was discharged in February 2024. During hospitalization, she developed corticosteroid‐induced adrenal suppression, which was used for her cerebral edema. An adrenocorticotropic hormone stimulation test confirmed this diagnosis. To address this, high‐dose dexamethasone was initiated and tapered gradually, and at the time of her visit to our clinic, she was receiving 10 mg of hydrocortisone daily.

### 1.1 Diagnostic Workup

Based on the patient's medical history and clinical presentation, a complication related to fat grafting was suspected. Atypical mycobacterial infection and deep mycosis were considered in the differential diagnosis. For definitive evaluation, two punch biopsies were obtained from the facial lesions and sent for PCR analysis (targeting the *rpoB* gene region for species identification), Ziehl‐Neelsen staining, hematoxylin and eosin (H&E) staining, and histopathological examination. In addition, a culture of the purulent discharge was performed.

**FIGURE 2:**
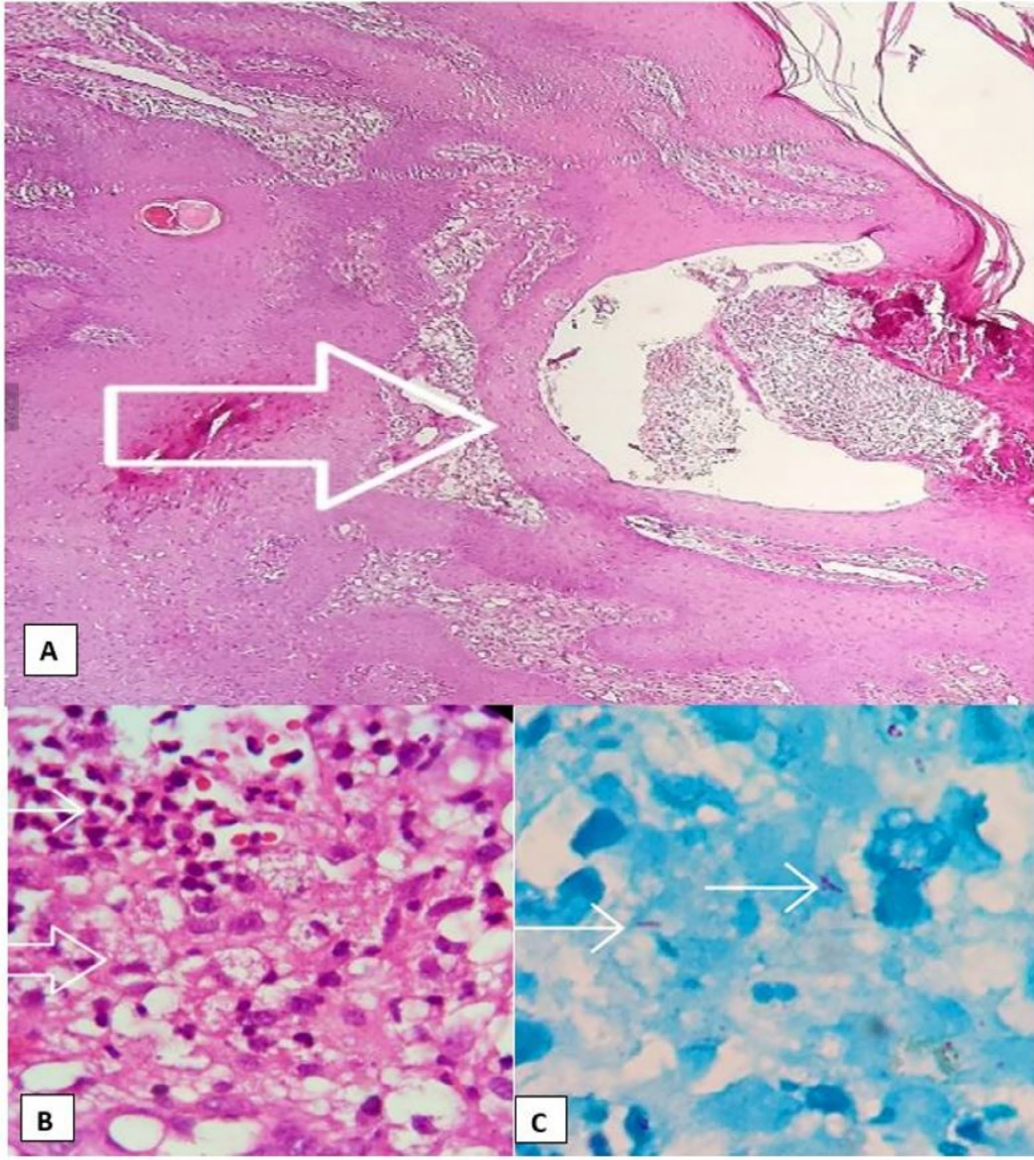
Histopathologic views: (A) Fistula tract filled with neutrophilic aggregates (arrow) (H&E, ×100). (B) Mixed dermal inflammation with granulomatous histiocytic aggregates (large arrow) and neutrophilic microabscesses (small arrow) (H&E, ×400). (C) Few acid‐fast bacilli visible on Ziehl–Neelsen staining (magnification ×1000, oil immersion).

Histopathologic examination revealed fistulous tracts with neutrophilic aggregates, granulomatous inflammation, and a few acid‐fast bacilli (Figure 2). Culture confirmed the presence of *Mycobacterium chelonae* ( *M. chelonae* ) in the skin lesions. Culture results were available within 5 days, consistent with the rapid growth pattern typical of *M. chelonae* .

### 1.2 Treatment and Outcome

An oral antibiotic regimen consisting of clarithromycin 500 mg twice daily (BID), minocycline 100 mg BID, and rifampin 300 mg BID was initially prescribed (Rifampin was included empirically at the start, before species confirmation, although *M. chelonae* is typically resistant to rifampin). This oral regimen was continued for 3 months; however, due to insufficient therapeutic response, the patient was subsequently hospitalized to initiate intravenous (IV) therapy. The IV regimen, administered for 1 month, included amikacin 500 mg daily, linezolid 600 mg BID, clarithromycin 500 mg BID, and imipenem 1 g BID, selected based on susceptibility patterns reported in the literature. Additionally, superficial skin abscesses were drained during the patient's hospitalization.

Laboratory investigations during admission revealed the following values: white blood cell count (WBC) 10 500/μL; hemoglobin (Hb) 12.5 g/dL; platelet count (PLT) 305 000/μL; fasting blood sugar (FBS) 105 mg/dL; blood urea nitrogen (BUN) 17 mg/dL; creatinine (Cr) 0.9 mg/dL; aspartate aminotransferase (AST) 32 U/L; alanine aminotransferase (ALT) 27 U/L; alkaline phosphatase (ALP) 155 U/L; total bilirubin (TBil) 0.4 mg/dL; direct bilirubin (DBil) 0.2 mg/dL; sodium (Na^+^) 138 mEq/L; and potassium (K^+^) 4.3 mEq/L.

After partial improvement of the inflammatory lesions, the patient was discharged on an extended course of oral therapy consisting of trimethoprim‐sulfamethoxazole (DS, BID), clarithromycin 500 mg BID, and moxifloxacin 400 mg daily. This oral phase was continued for 6 months. At 6 months of follow‐up after discharge, evaluations have shown no evidence of disease recurrence or further complications; however, disfiguring scars on her face still remain and represent another therapeutic challenge, possibly requiring surgery and resurfacing after complete clearance of the infection.

Given the severity of the infection and the complications that developed in this patient, we found it essential to contextualize this case within the broader spectrum of non‐tuberculous mycobacterial (NTM) infections reported as complications of cosmetic procedures, in order to compare its clinical presentation, course, and treatment strategy with the current body of evidence. The following literature review summarizes existing reports of NTM infections following fat grafting, focusing on their clinical course, outcomes, and treatment strategies.

## 2 Review of Literature

The global increase in aesthetic procedures has led to a corresponding rise in their complications, including rare but severe infections by NTM, especially when performed in unregulated environments. To date, there is no dedicated epidemiologic study estimating the incidence of NTM infections following fat grafting. However, one study indicated that 17.5% of all NTM infections were caused by unsterile cosmetic injections, with clarithromycin, followed by amikacin, being the most frequently used antibiotics for their treatment [1, 2].

In this article, we review cases of NTM infections following autologous fat grafting, which was done by searching PubMed for case reports and case series published between 2015 and 2025 using the keywords ‘fat grafting’ and ‘mycobacteria’. We identified 16 reported cases of NTM infection related to fat grafting from different countries. Although no epidemiologic studies quantify the exact incidence of these complications, most cases suggest an association with procedures performed in non‐sterile environments and by non‐specialists. These infections have been documented following fat grafting to various anatomical sites; the face was the most frequently affected anatomical site, followed by the buttocks, breasts, and extremities. Approximately three‐quarters of cases were female, and the ages ranged from 23 to 65. In most cases, the pathogen was rapidly growing mycobacteria (RGM), particularly *Mycobacterium abscessus* , *M. chelonae* , *M. fortuitum* , and *M. mageritense* . Four cases were linked to combined procedures (e.g., abdominoplasty with fat transfer), and 2 were associated with cryopreserved fat injection, both of which can increase the risk of contamination (Table 1) [3, 4, 5, 6, 7, 8, 9, 10, 11, 12, 13].

**TABLE 1:** Reported cases of atypical mycobacterial infections following autologous fat grafting and related procedures.

Case	Sex	Age	Country	Site	Procedure	Culture
1	F	35	Kurdistan	Hands, feet, breasts	AFG	*M. abscessus* [3]
2	F	39	Germany	Face	AFG	*M. fortuitum* [4]
3	F	23	Dominican Republic	Buttocks	AFG	*M. abscessus* [5]
4	F	53	Brazil	Buttocks	Abdominoplasty, liposuction, and AFG	*M. abscessus* [6]
5	F	46	Brazil	Abdomen	Abdominoplasty, umbilical herniorrhaphy	*M. fortuitum* [6]
6	F	65	Brazil	Buttocks	Abdominoplasty, liposuction, and AFG	*M. abscessus* [6]
7	M	35	Brazil	Shoulder	AFG	*M. conceptionense* [6]
8	F	25	Iran	Face	Cryopreserved AFG	*M. mageritense* [7]
9	F	27	Iran	Face	Cryopreserved AFG	*M. mageritense* [7]
10	F	46	China	Breast	Liposuction of the thigh and subsequently AFG for breast augmentation	*Corynebacterium bovis* [8]
11	F	38	Korea	Face	AFG	*M. chelonae* [9]
12	F	48	Korea	Face	AFG	*M. chelonae* [9]
13	F	29	China	Face	AFG	*M. abscessus* [10]
14	F	35	Korea	Face	AFG	*M. fortuitum* [11]
15	F	40	Taiwan	Face	AFG	*M. abscessus* [12]
16	F	59	USA	Face, Periocular	AFG	*M. chelonae* [13]

Abbreviations: AFG, autologous fat grafting; F, female; M, male; M., Mycobacterium; USA, United States of America.

The clinical presentation varies from localized cellulitis to osteomyelitis. NTM infections should be considered in patients presenting with delayed, atypical, inflammatory, and purulent skin lesions following procedures [14] Diagnosis can be confirmed through acid‐fast staining, mycobacterial culture, and PCR [15]. Treatment typically involves prolonged multi‐drug regimens, often including clarithromycin, amikacin, or imipenem, and surgical interventions such as lesion drainage or debridement. Emphasis should be placed on proper sterilization techniques to prevent such infections [16].

As most of the data on this subject comes from case reports and case series, future research is warranted to determine the incidence of these complications and to identify effective treatment protocols, particularly in light of the growing problem of antibiotic resistance and its variable patterns across different countries.

Compared to most of the reported cases, our patient experienced a more prolonged clinical course, which required prolonged multidrug antibiotic treatment, highlighting the variability in NTM susceptibility and the importance of region‐specific antimicrobial data. Most previously reported cases did not experience any systemic involvement. In contrast, our patient developed severe systemic complications, including cerebral edema and coma, making this case exceptional among reported *M. chelonae* infections following fat grafting.

## 3 Discussion

Autologous fat grafting is a widely used technique for facial rejuvenation and volume enhancement, aimed at mitigating the signs of facial aging. Generally, autologous fat grafting is considered a safe treatment method [17]. However, several side effects have been reported due to the rising number of procedures performed recently. These include acute cerebral infarction, vision loss, epidermal cysts, as well as redness, swelling, bruising, vascular occlusion, necrosis, hypersensitivity reactions, infections, and foreign body reactions [18, 19]. To our knowledge, this is among the first reported cases of *M. chelonae* infection following unauthorized facial fat grafting that progressed to cerebral complications and adrenal suppression.

Schiraldi and colleagues reported an incidence of 7.7% (354 out of 4579) for complications in their meta‐analysis. The most common adverse effects in this study were intravascular injections (24.6%), protracted edema (20.0%), irregularities at the injection site (16.1%), activation of acne (14.1%), fat necrosis or lipogranuloma (8.2%), graft hypertrophy (6.2%), telangiectasia (2.4%), asymmetry (3.7%), prolonged erythema (1.7%), and infection (0.3%) [20].

Autologous fat grafting has recently become common in cosmetic clinics. However, non‐tuberculous mycobacterial infections, especially with RGM species following this procedure, have also been reported [21]. Rapidly growing mycobacteria (RGM) species are *Mycobacterium abscessus* , *M. chelonae* , and *M. fortuitum* . Their high prevalence in the environment and resistance to common sterilization procedures make them a difficult pathogen to eliminate [22]. The latency of this infection usually leads to some delays in the diagnosis. Persistent painful, erythematous lesions with swelling unresponsive to usual treatment following cosmetic procedures such as fat transfer could imply the possibility of this diagnosis [23] The management of our patient, which began with clarithromycin and minocycline and then proceeded to amikacin, imipenem, clarithromycin, and linezolid due to lack of response, reflects a major challenge that clinicians face in practice when treating NTM infections. In some cases, surgical debridement is required to achieve complete eradication of NTM infection, particularly in multifocal or resistant cases [22] This challenge is due to both the intrinsic resistance of this species to many conventional antibiotics and the increasing emergence of acquired resistance. The antibiotic susceptibility pattern of NTM varies across different geographic regions. As shown in a systematic review by Nejad et al. published in the Journal of Mycobacteria in 2025, in Iran, *M. chelonae* , like many other NTMs, shows the highest susceptibility to amikacin, which was also true in our present case. In most other areas of the world, the most susceptible drug for treating *M. chelonae* is tobramycin. This highlights the need for regional and epidemiologic data to better understand the antibiotic susceptibility patterns of NTM in different areas. The emergence of resistant NTM species underscores the importance of avoiding unnecessary antibiotic use [24].

These complications emphasize that clinicians performing cosmetic procedures must not only master technical skills but also maintain strict infection control and recognize atypical postoperative infections. Coherent oversight structures must be in place to ensure that cosmetic procedures are performed by qualified specialists.

This case emphasizes the paramount importance of sterility in fat grafting procedures. Strict aseptic techniques, sterile handling of harvested fat, along with the avoidance of cryopreserved or reused fat, can markedly reduce the risk of NTM contamination. Furthermore, comprehensive regulation and continuous staff training in infection control are essential to prevent similar complications.

## 4 Conclusion

Clinicians should maintain a high index of suspicion for NTM infection in cases of delayed, non‐healing post‐procedural inflammation unresponsive to conventional antibiotics. Tailored therapy based on culture or local resistance data, along with strict infection control regulations, is essential to prevent these potentially devastating complications.

## Author Contributions

M.I.: data curation, conceptualization; F.B.: data curation, histopathology evaluation; I.A.D.: data curation, microbial evaluations and treatment planning; R.M.R.: conceptualization, writing the draft, editing, supervision; S.Z.: writing and editing the manuscript.

## Funding

The authors have nothing to report.

## Ethics Statement

Reviewed and approved by the ethics committee supported by the deputy of research and technology at Shahid Beheshti University of Medical Sciences, Tehran, Iran, with code number IR.SBMU.SRC.1403.041.

## Consent

The patient in this manuscript has given written informed consent to the publication of their case details.

## Conflicts of Interest

The authors declare no conflicts of interest.

## Data Availability

The data that support the findings of this study are available on request from the corresponding author. The data are not publicly available due to privacy or ethical restrictions.
